# Angiomatoid fibrous histiocytoma (AFH) unusual clinical presentation and unique radiological findings

**DOI:** 10.1259/bjrcr.20190069

**Published:** 2020-12-02

**Authors:** Mohammed Khader, Tahiya Alyafei, Sulafa Ibrahim, Orwa Elaiwy

**Affiliations:** 1 Department of Radiology, Hamad General Hospital, Doha, Qatar; 2 Department of Pathology, Hamad General Hospital, Doha, Qatar

## Abstract

Angiomatoid fibrous histiocytoma (AFH) are rare soft tissue mesenchymal neoplasms that commonly affect children and young adults. They are classified as “intermediate tumours of uncertain differentiation”. We present a case of an 8-year-old child with a left thigh AFH and antecedent history of minor trauma showing perilesional oedema and enhancement at MRI, leading to an initial working diagnosis of infected haematoma that contributed to the challenge in reaching the final diagnosis. Although most of the imaging features of AFH previously described in the literature are demonstrated in this case, the presence of arterial vascular channels within the tumour and feeding branch from the left profunda femoris artery is unusual and to our knowledge the first to be published in the literature.

## Case presentation

An 8-year-old Yemini male with no significant past medical history presented with dark skin discolouration overlying a slow-growing mobile subcutaneous mass in the mid-lateral left thigh over 6 months. The lesion appeared after suspected trauma with an unknown object (possibly a nail or piece of wood). Growth was most marked over the preceding 2 months. The patient had intermittent low-grade fever, usually at night, with weakness and fatigue. Examination revealed dark brownish skin discolouration (approximately 5×5 cm), with a central hypopigmented area where the skin scaled off and underlying mobile subcutaneous firm non-tender swelling feeling mildly warm to touch. Initial laboratory findings revealed low haemoglobin level of 7.9 gm dl^−1^, elevated C- reactive protein of 102 mg l^−1^, elevated erythrocyte sedimentation rate of 42 mm/h with normal differential WBC count.

## Investigations

**Figure 1.**
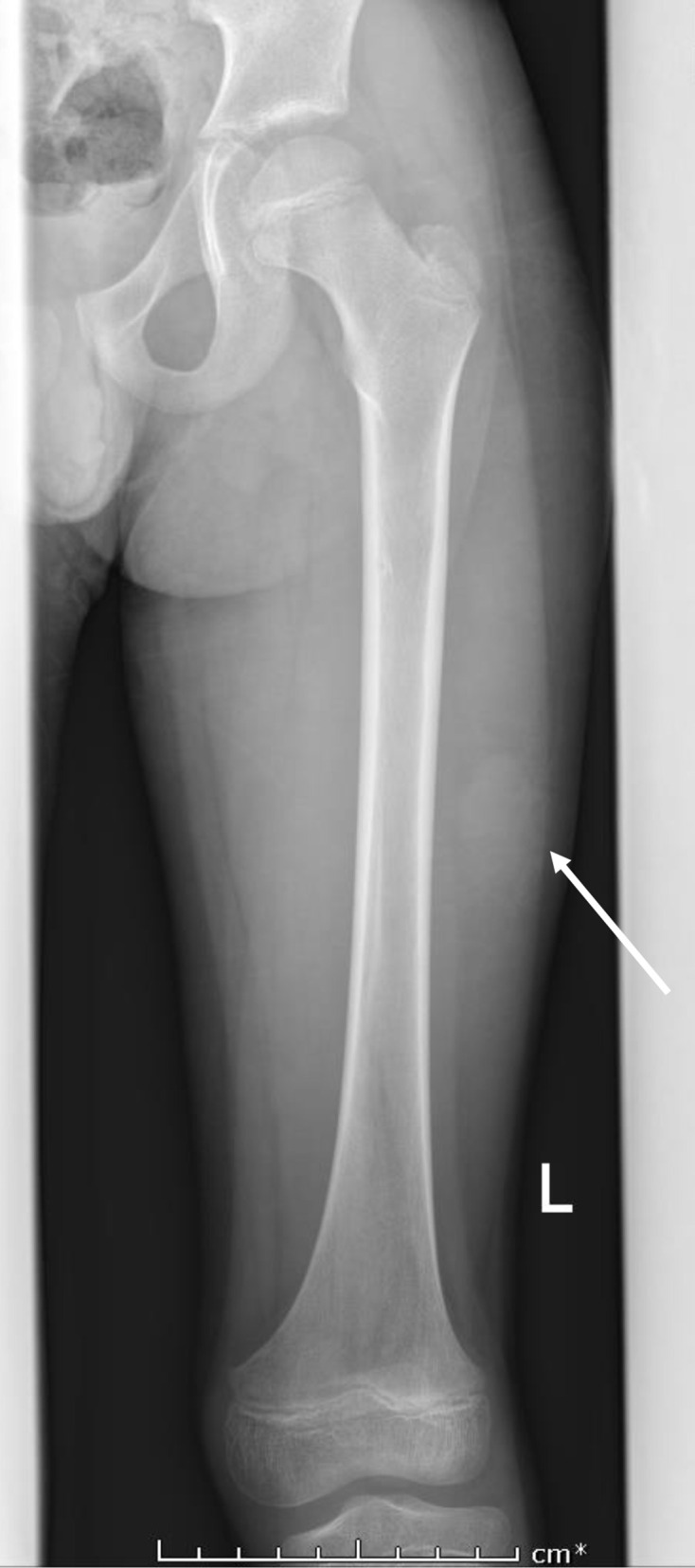
8-year-old male with angiomatoid fibrous histiocytoma. Anteroposterior radiograph of the right femur demonstrating an ovoid soft tissue lesion in the mid-left thigh (arrow).

**Figure 2.**
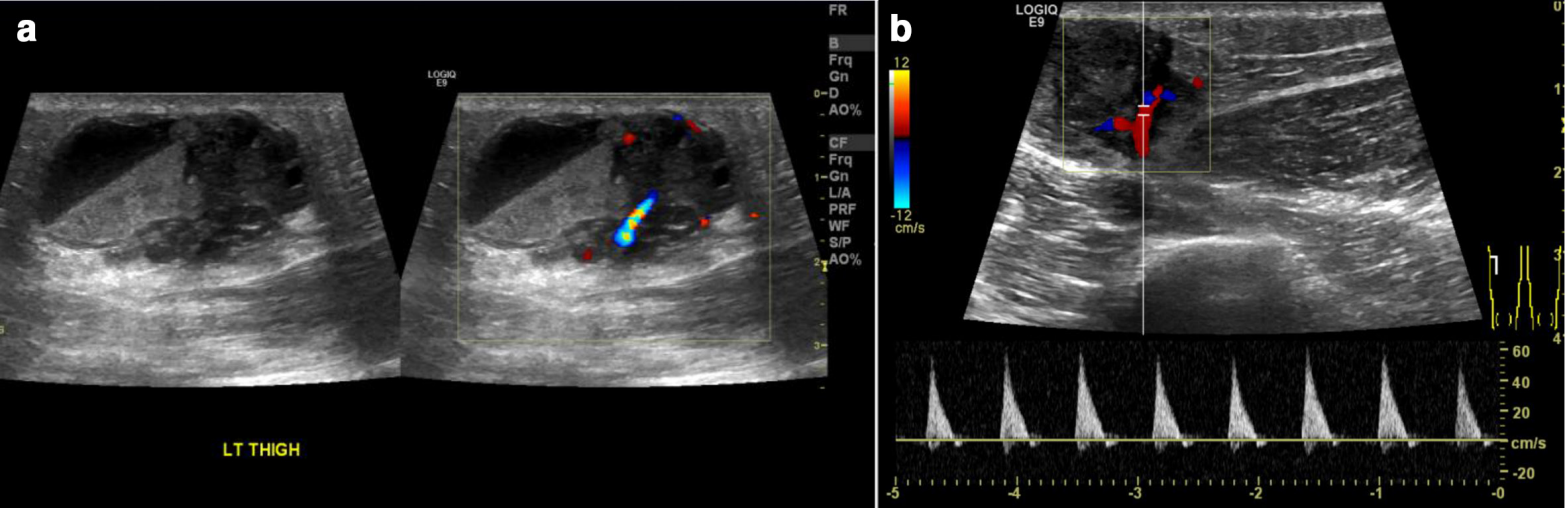
Ultrasound examination of AFH (a) Composite grayscale and colour Doppler imaging shows a heterogeneous complex mainly cystic lesion with fluid-debris levels (likely blood-fluid levels). (b) Spectral Doppler imaging shows intralesional vascular channels with high-resistance arterial spectral waveforms.

**Figure 3.**
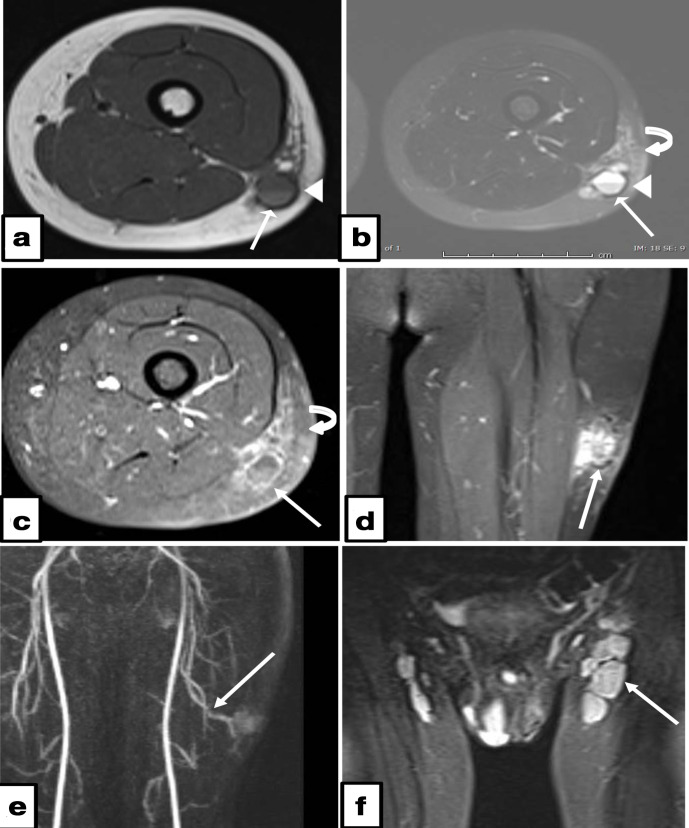
MRI of AFH. (a) Axial T1W and (B) axial T2 fat suppressed imaging shows a 3.4×2.3×2.6 cm heterogeneous ovoid subcutaneous lesion in the fat of the lateral aspect of the mid-left thigh without evidence of muscle infiltration (arrow). The lesion is isointense to muscle on T1WI with fluid-fluid level (arrowhead), and heterogeneously hyperintense on T2 fat saturation. The periphery of the mass demonstrates the typical low signal on non-enhanced images representing a fibrous pseudo capsule (arrows). (c) Axial T1 post gadolinium showing peripheral enhancement (blue arrow), with surrounding peritumoural soft tissue edema and enhancement noted (curved arrow in b & c). (d) Coronal T1 post-contrast imaging depicting variegated pattern of intralesional enhancement. (e) Static TWIST coronal MRA imaging of the left thigh with a prominent arterial branch from left profunda femoris artery noted to directly supply the tumour (yellow arrow). (f) Coronal STIR image of the inguinal regions with prominent ipsilateral inguinal lymph nodes (arrow).

Plain radiographs demonstrated subtle soft tissue opacity in the lateral aspect of the left mid-thigh with no radiopaque foreign body or soft tissue gas (Figure 1). Ultrasound examination demonstrated a complex mainly cystic lesion measuring 3.6×2.4×1.9 cm, with interspersed vascular channels showing arterial spectral Doppler waveforms (Figure 2) and occasional fluid-fluid levels. MRI examination (Figure 3) depicted the mass to be heterogeneously high signal on T2 fat-sat and isointense to muscle on T1W imaging with blood product-fluid levels and peripheral low signal on non-enhanced imaging representing a fibrous pseudocapsule. The lesion showed areas of central intense enhancement and peripheral variegated pattern of enhancement. Perilesional soft tissue oedema and enhancement was also appreciated. A large feeding artery supplying the lesion, a terminal branch of left profunda femoris artery and enlarged ipsilateral inguinal lymph nodes were also noted.

**Figure 4.**
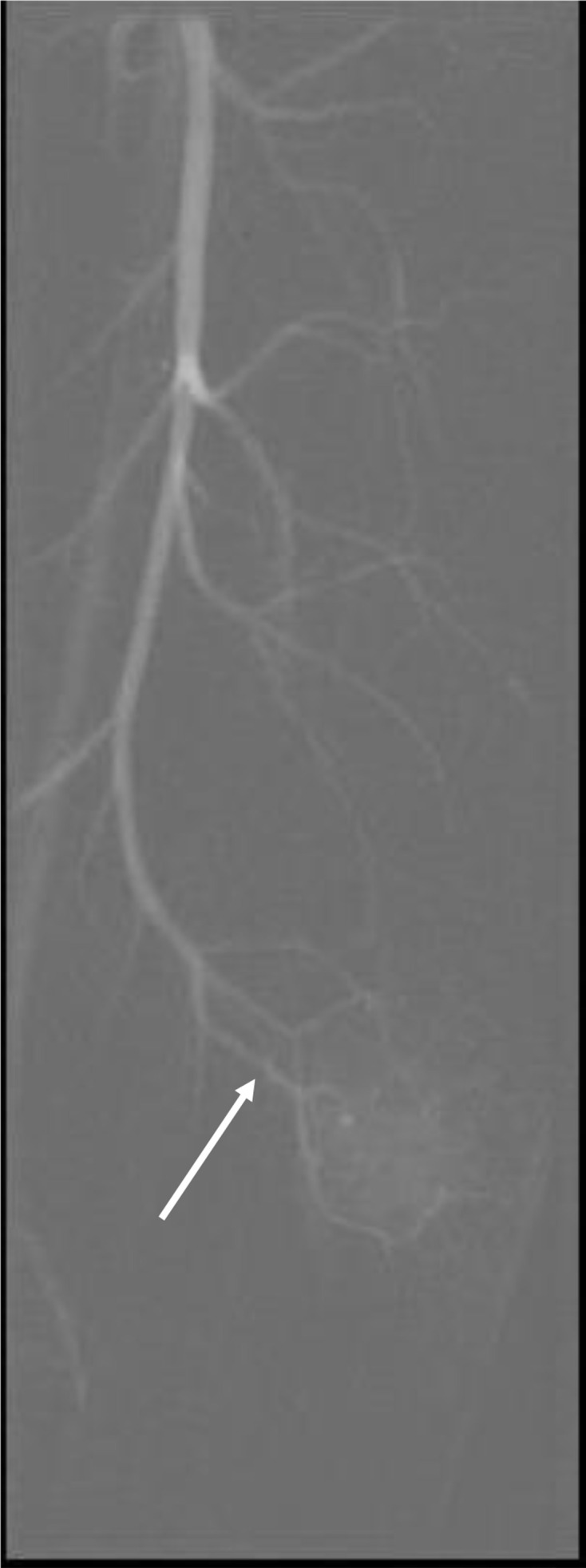
Conventional digital subtraction angiogram of left lower limb showing a vascular lesion in lateral aspect of the mid left thigh supplied by a prominent terminal branch of the profunda femoris artery (arrow). No evidence of nidus or arteriovenous malformation.

The initial differential diagnosis was post-traumatic infected haematoma with underlying vascular malformation or vascular tumour not excluded. The patient was followed closely and given antibiotics with only minimal improvement of the local symptoms. Follow-up ultrasound after 2 weeks revealed no significant interval change. Left femoral digital subtraction angiogram was performed and confirmed the presence of the vascular lesion in the left mid-thigh along the lateral aspect supplied by a prominent branch arising from the terminal part of profunda femoris artery (Figure 4); however, there was no evidence of nidus or arteriovenous malformation.

Ultimately, the patient underwent surgical resection with gross specimen appearances showing a solid non-haemorrhagic lesion 3×3 cm attached to tensor fascia lata of left lateral thigh, and no infiltration to underlying muscles or bone, the vascular pedicle was posterior and intact.

**Figure 5.**
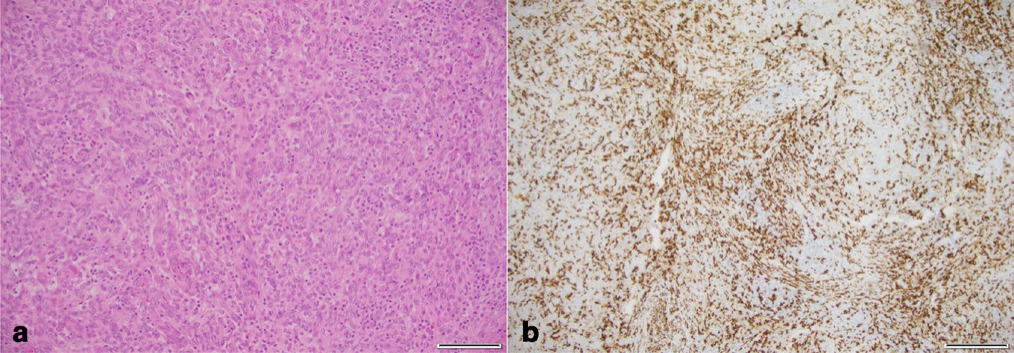
AFH tumour histopathology. (a) H-E stain (x200) and (b) CD68 immunostain (x200). (a) The resection specimen demonstrates areas of spindled cells and microscopic haemorrhage resembling vascular spaces with peripheral lymphoplasmacytic infiltrate and germinal centres within a dense hyaline fibrous pseudocapsule confirming a diagnosis of AFH. (b) Diffuse positivity for CD68 immunostaining.

Histopathological examination (Figure 5) revealed a lobulated tumour with epithelioid histiocytic foci and other areas with spindle cells arranged in storiform distribution. It contained a few angiomatous spaces with evidence of both recent and old haemorrhage. The tumour was surrounded by a fibrous pseudocapsule with prominent lymphoid infiltrate. In areas, the tumour had an almost angiomatous appearance with tight whorls. The tumour is infiltrated by a mixture of lymphocytes, plasma cells and macrophages often containing haemosiderin.

Cranial MRI and staging contrasted CT of the chest, abdomen and pelvis performed confirmed no evidence of metastasis or satellite lesion. Repeat thigh MRI 1 month after resection demonstrated minimal postoperative oedema and seroma without evidence of residual lesion. Ipsilateral prominent inguinal lymph nodes were re-demonstrated. Selective excisional nodal biopsy was performed showing follicular hyperplasia with no evidence of malignancy.

## Discussion

AFH is a rare soft-tissue mesenchymal neoplasm of uncertain histogenesis and line of differentiation that most commonly affects children and young adults. AFH was described initially as “angiomatoid malignant fibrous histiocytoma” by Enzinger in 1979. Currently, it is no longer regarded as “malignant” because of its benign microscopic appearance and favourable prognosis. In the 2013 World Health Organization (WHO) classification, AFH are classified as “intermediate tumours of uncertain differentiation”.^1,2^

No well-organized clinical reports exist for AFH because it is a very rare soft tissue tumour. AFH often presents as a slowly growing painless mass in the deep dermis or subcutis in children and young adults with a median age of 13 years but may also occur in middle-aged patients. Some tumours invade deep structures such as skeletal muscle and rarely in other sites such as the lung and bone.^3–5^ A small proportion of patients experience systemic signs and symptoms including pyrexia, anaemia, and malaise suggesting tumoural cytokine production. Symptoms of pain and tenderness are rarely encountered.^6^ In this case, the clinical presentation with suspected history of trauma was misleading, leading to a working diagnosis of infected haematoma, and it contributed to the challenge of reaching the final diagnosis.

More than 400 cases of AFH have been reported in the pathology literature. By comparison, the imaging features of AFH are less well established. A few series of well-documented cases of soft tissue AFH with comprehensive accompanying MRI and several with limited MRI have been published in the radiology literature. AFH typically occur in the extremities but may occur anywhere in the body.^6–15^

Clinical and radiological differentiation of AFH from haematoma or vascular tumours such as haemangioendothelioma and angiosarcoma is challenging. The imaging findings of AFH are often non-specific. When originating in the soft tissues, radiographs are of limited value and contrast-enhanced computed tomography with its limited soft tissue contrast resolution may show a heterogeneous mass, and possibly hint at cystic and enhancing components.^6,16^

US and MRI are preferred to evaluate soft-tissue masses. US findings in AFH have been described in very few cases. One case report in a 77-year-old female with a temporal region lesion showed an irregular-shaped hypoechoic cystic lesion with peripheral flow, US elastography revealed high elasticity centrally.^17^

A case series of four children undergoing US showed a predominantly solid appearing mass in three of which there were tiny cystic changes in two, and in one the mass had a multi-cystic appearance with fluid-fluid levels of mixed echo-texture with septal and peripheral colour Doppler flow.^9^

In our case, the latter described sonographical findings were also noted in addition to the presence of vascular channels showing arterial spectral flow pattern which is to best of our knowledge have not been previously reported in literature for histopathologically proven AFH.

Some MRI features of AFH previously described in the literature may help in the diagnosis although none are considered specific, the most helpful finding is the presence of intralesional cystic spaces with fluid-fluid levels. In general, AFH show homogeneously isointense T1 signal and heterogeneously hyperintense foci on T2W imaging, with peripheral nodular and variegated internal pattern of gadolinium enhancement. Additional reported features include the presence of a pseudocapsule, haemosiderin and in some cases perilesional oedema.^6–10,12,15,16,18^ The presence of a rim of high signal intensity and adjacent inner rim of low signal intensity which can be observed on both T2W and post-contrast images, referred to as the ‘double rim sign’ is considered a novel-specific sign for AFH.^15^ The double rim may be partial or not clearly delineated as in this case where only a peripheral low signal rim was seen representing a fibrous pseudocapsule.

A unique imaging finding which was not previously described as features of AFH was present in this case, which is the presence of a feeding arterial branch directly to the lesion. Conventional angiography excluded the presence of AV malformation and revealed blood supply to the lesion as a prominent terminal branch of the profunda femoris artery (Figure 4).

MRI is indicated for local staging. Accurate staging of nodal and distant metastases is mandated, fluorodeoxyglucose positron emission CT (FDG PET-CT) has been used for staging but caution is required in interpreting nodal involvement as biopsy of suspicious nodes seen on PET-CT have subsequently been found to be benign on histopathological analysis.^19^ Nodal metastases are uncommon from soft tissue neoplasms.^20^

The lesion in our patient showed the typical histopathological findings of AFH, which have been well-described in the literature, including fibrous pseudocapsule, peritumour lymphoplasmacytic infiltrate, cystic spaces containing blood products surrounded by tumour cells and stromal haemosiderin deposition, and aggregates of histiocytoid and spindle-shaped tumour cells in a whorled or fascicular distribution.^9^

The management of AFH includes surgical wide local excision with imaging surveillance for recurrence. With unrespectable or metastatic disease, adjuvant chemotherapy and/or radiation therapy may be used.^21^

The prognosis of patients AFH does not appear poor; however, local soft tissue recurrence is seen in up to 15% of cases with metastases in under 1%.^16^

## Learning points

- Angiomatoid fibrous histiocytoma (AFH) is a rare soft tissue tumour with intermediate malignant potential that most commonly affects children and young adults. It usually occurs as a painless mass in an extremity but can occur anywhere.
- We present a case of AFH with misleading antecedent history of trauma, initially leading to a working diagnosis of infected haematoma which contributed to the challenge of reaching the final diagnosis on diagnostic imaging alone.
- While the imaging features of AFH are not well established in the literature and typically non-specific, a mass with cystic areas, pseudocapsule, internal fluid-fluid levels and variable enhancement in the extremity of a child or adolescent should prompt the consideration of AFH in the differential diagnosis. A unique imaging feature was present in this case, the presence of a feeding arterial branch directly to the lesion, which is to the best of our knowledge, the first to be published in literature.
